# Effects of* Camellia Sinensis* Extract on Repeated Restraint Stress-Induced Ovariectomized Female Rats

**DOI:** 10.1155/2019/1926352

**Published:** 2019-07-24

**Authors:** Minsook Ye, Daehyuk Jang, Jin Su Kim, Kyungsoo Kim, Insop Shim

**Affiliations:** ^1^Department of Biomedicine & Health Sciences, College of Medicine, The Catholic University of Korea, 222, Banpo-daero, Seocho-gu, Seoul 06591, Republic of Korea; ^2^Department of Physiology, College of Medicine, Kyung Hee University, Seoul 02435, Republic of Korea; ^3^Molecular Imaging Research Center, Korea Institute of Radiological & Medical Sciences University of Science & Technology, #215-4 Gongneug-dong, Nowon-ku, Seoul 139-241, Republic of Korea; ^4^Department of Family Medicine, College of Medicine, The Catholic University of Korea, 222, Banpo-daero, Seocho-gu, Seoul 06591, Republic of Korea

## Abstract

The mortality of individuals suffering from depression has been increasing, noticeably of postmenopausal women; consequently, their care and treatment are significant to retain a high quality of life. The aim of this study was to examine the effect of* Camellia sinensis* (CS) on repeated stress-induced changes of the depression related function on the tail suspension test (TST), forced swimming test (FST) in ovariectomized female rats. After behavioral test, we evaluated the changes in the neurotransmitter by measuring the level of dopamine in the nucleus accumbens (NaC) and the serum levels of estrogen and oxytocin. We used 18F-2-fluoro-deoxy-D-glucose positron emission tomography (18F-FDG-PET) to examine the effects of CS on glucose metabolism in ovariectomized rats. Female rats were randomly segregated into three groups. Nor group was considered as nonoperated and nonstressed group, while the control was the ovariectomized and stressed group (OVX+ST), and CS was the ovariectomized, stressed and CS treated group. The rats were exposed to immobilization stress (IMO) for 14 d (2 h/d), and CS (300 mg/kg, i.p.) was treated 30 min before IMO stress. Significant reduction of immobility in the TST and FST was indicated in rats treatment with CS compared to the control group (OVX+ST). The levels of estrogen in the serum of the Nor and CS groups were significantly elevated compared to the OVX+ST group. Also, CS activated brain glucose metabolism in the cortex. The present findings suggested that CS had antidepressant effectiveness in a menopausal depression animal model. These findings suggest evidence that CS plays a crucial role in stressful situation, providing that CS might be a dependable antidepressant medicine to treat menopausal depression.

## 1. Introduction

Depression is the most common of all the psychiatric disorders, and it is the main clinical manifestation of menopausal depression. In the National Comorbidity Survey, major depression is more ordinary in women than in men until late life, with a lifetime prevalence of 21% compared to 12% for men [1]. It was found that estrogens have intense effect on symptoms of depression [2]. A reduction in estrogen levels is related to aroused sensibility to develop major depressive disorder (MDD) [3]. Also, rodents have shown an increase in depression-like behavior during the diestrus phase of the estrous cycle when the estrogen levels are low [4]. Ovariectomized (OVX) animal models of estrogen lack have symptoms similar to those of women with menopause-related depression [5]. Recent study has placed emphasis on herbal medicines for development of antidepressant drug. The polyphenols have taken continuously more interest in supplementary interventions to preserve health and treat diseases [3, 4]. Polyphenols have a broad spectrum of biological activities such as neurodegenerative disorders, cancer, and cardiovascular disease, and cardiovascular diseases contains the regulation of cell growth and proliferation, enzyme activity, and the modulation of cellular signaling cascades [1].

*Camellia sinensis* (CS) is one of the most popular beverages in the world. Several studies suggest that green tea has many health advantages, such as the antistress, anticancer, and antioxidant effacement [6]. CS is becoming more popular throughout the world for its beneficial effects such as vasodilatation, advanced plasma lipid profile, and elevated insulin sensitivity [1]. Being rich in flavonoids, it is a potential candidate for the treatment of cancer, obesity, Parkinson's, stress, depression, and related disorders. The main catechins in CS leaves are (-)-epigallocatechin gallate ((-)-EGCG), (-)-epigallocatechin ((-)-EGC), (-)-epicatechin gallate ((-)-ECG), and (-)-epicatechin ((-)-EC). Tea catechins undergo epimerization during the course of the manufacturing and brewing processes [1]. In fact, when canned and bottled tea drinks were pasteurized at 120°C for several minutes, considerable amounts (around 50%) of catechins were epimerized at the 2-position and (-)-catechin (C), (-)-gallocatechin (GC), (-)-catechin gallate (CG), and (-)-gallocatechin gallate (GCG) were formed. The concentration of these epimers present in canned and bottled tea drinks was similar to or even greater than that of green tea catechins [7]. However, studies on physiological effects of heat-epimerized catechins are scarce. For these reasons, we hypothesized that CS might play a critical role in the treatment of depression. Previous studies suggested that CS possesses therapeutic effects in the immobilization stress-induced stress model [8]. Tricyclic antidepressants (TCAs) are antidepressant medicines that interact having biological effects and similar chemical structure. Tricyclic antidepressants ascend norepinephrine and serotonin level and inhibit the action of acetylcholine, another neurotransmitter. Several studies suggest that by restoration of the balance in these neurotransmitters in the brain, tricyclic antidepressants mitigate depression. Since many side effects due to chronic administration limit the therapeutic treatment, it is necessary to unveil new targeted medicine with the claim of a more favorable tolerability and efficacy profile [9]. Despite years of study, the mechanisms that trigger the disease are not entirely conquered. Consequently, the confirmation of effective and safe treatments with evident pharmacological mechanisms is wanted.

The monoamine hypothesis founded on the lack of monoamines is generally aroused to describe the physiopathology of depression. This hypothesis found on lack of noradrenaline, serotonin, and dopamine (DA) [10]. The link between DA and depression was corroborative by the evidence that antidepressants act on the dopaminergic system. Whereas basic and clinical studies suggest lack of the dopaminergic system in depression, the origin of these deficits likely lies in dysregulation of its regulatory afferent circuits. Researches on animal models insist that antidepressants improve neurotransmission in the dopamine mesolimbic system [1].

Diminished glucose metabolism of prefrontal cortex was reported to be significantly correlated with depression severity in people with major depressive disorder [1]. To test this hypothesis, we used [18F] fluorodeoxyglucose (FDG) micro-positron emission tomography (PET) to determine whether CS increases glucose in a manner close to that of antidepressants in the brain [11].

In the present study, we examined the effect of CS on the stress and depressive-like behavior of ovariectomized rat using the tail suspansion test (TST) and forced swimming test (FST), which are useful for investigation of antidepressants compound [12]. Furthermore, we investigate concentration of DA in the nucleus accumbens (NaC) and of estrogen and oxytocin in serum using the ELISA as a neurobiological measure. In addition, we have tried to assess brain glucose metabolism changes with [F-18] FDG micro PET in rats.

## 2. Material and Methods

### 2.1. Regents

The high temperature processed-green tea extracts (HTP-GTE) were extracted and HPLC-PDA protocol was performed as described in the previous study [13]. Fresh green tea (*Camellia sinensis*, CS) leaves were gathered in spring from Osulloc Tea Garden in Jeju, Korea, and were dried at 150°C for 10 min. The dried CS leaf was 50% aqueous ethanol extracted at 60°C for 3 h by using water bath. The 50% aqueous ethanol extract was dissolved with water and then processed at high temperature for epimerization of catechins. HTP-GTE was concentrated with a rotary evaporator (Buchi R200, Flawil, Switzerland) in vacuo and stored at −20°C for use (Table 1). HTP-GTE was prepared and provided by Amorepacific Corporation R&D unit.

For the phytochemical analysis of fermented* Camellia sinensis* (CS) leaves acetone-soluble extract using HPLC-PDA a Zorbax C18 column (250 × 4.6 mm, I.D., 5 *μ*m; Agilent Technologies) was conducted. The mobile phases for chromatography consisted of acetonitrile for solvent A (acidified by 0.01% trifluoroacetic acid) and water (acidified by 0.01% trifluoroacetic acid) for solvent B. The mobile phase gradient was 5% A (0–5 min), 5%–30% A (5–30 min), 30%–100% A (30–50 min), and 100% A (50–60 min). The mobile phase flow-rate was 1 mL/min. The injection volume for all samples was 20 *μ*L. The HPLC chromatogram was monitored at UV 280 nm (Figure 2).

### 2.2. Animals and Treatment

**Figure 1:**
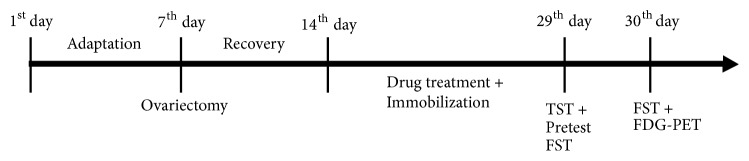
Experimental schedule of OVX-induced depression-like behaviors and treatment with CS in rats.

**Figure 2:**
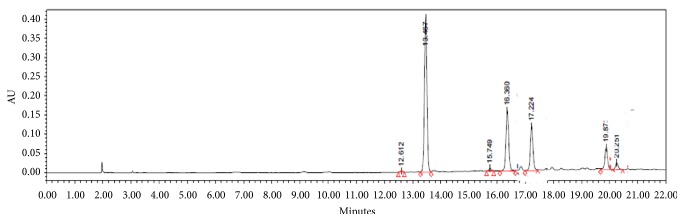
HPLC chromatogram of the ethanol-soluble extract of high temperature processed-green tea extract (HTP). Peaks 1: caffeine, 2: (−)-epigallocatechin 3-O-gallate, 3: (−)-gallocatechin 3-O-gallate, and 4: (−)-epicatechin 3-O-gallate, 5: (+)-catechin 3-O-gallate.

Sprague Dawley female rats of 7-week-old from the Samtako Animal Co. (Seoul, Korea) were used for this experiment. The rats were kept under a regulated temperature (22-24°C) with 12 hours light/dark cycle (lights on at 8:00 and off at 20:00). They had standard diet and water until being sacrificed. This experiment was executed in accordance with the National Institutes of Health* Guide for the Care and Use of Laboratory Animals *revised in 1996 and was approved by the Institutional Animal Care and Use Committee of KyungHee University (KHUAP(SE)-13-041). The grouping was split into the following at random: the nonoperated and nonstressed group (Nor), the ovariectomized and stress group (OVX+ST), the ovariectomized, stressed and treated with (CS) 300 mg/kg group (CS 300). The ovariectomy was operated under pentobarbital sodium (50 mg/kg, i.p.). After recovery for 1 week, the rats were stressed daily except Nor group. The rats were forced into an immobilizer device (a disposable rodent restraint cone) to get stressed out during 2 hours (13:00–15:00) for 2 weeks. The Nor and OVX+ST group was orally administrated sterile saline and other groups were each given extract appropriate doses once a day for 2 weeks. Administration of drug began 30 min earlier than the immobilization stress (Figure 1).

### 2.3. Tail Suspension Test

After 14th days of the immobilization, rats underwent a TST. The TST was performed based on the method by Steru [14]; the total time immobile induced by tail suspension was measured. Briefly, a rat was placed such that one's head was about 10 cm above the floor in each chamber. Animals taping their tail approximately 1 cm from the end were suspended to the edge of a lever below chamber roof.

### 2.4. Forced Swimming Test

After 14th days of the immobilization, rats were conducted an FST. The FST transparent Plexiglas cylinder (20 cm diameter, 50 cm height) was used in FST. At the testing room temperature, the cylinder was filled up with water to 30 cm depth so that no rat could touch the bottom with their tails. At 14th day, all of groups for examination were trained for 15 min by placing them for training to the cylinder. At 15th day, rats were subjected to 5 min of forced swim and scored the duration of immobility, swimming, and climbing. Climbing was indicated as upward-directed movements of the forepaws along the side of cylinder and swimming was known as movement throughout the chamber. Immobility was calculated as the length of time meant to perform the minimum movement necessary to stay float [15].

### 2.5. ELISA Analysis

After PET scan, the animals were anaesthetized. Blood was collected quickly via intracardiac puncture to determine estrogen and oxytocin concentration. Collected serum from the blood and gathered tissue from brain were stored at −20°C until being used. Using estrogen ELISA kit and oxytocin ELISA kit, each concentration of estrogen and oxytocin in serum was measured by Rat Estrogen ELISA kit, Abbexa, Cambridge, UK and Oxytocin ELISA kit, Enzo Life Sciences, Inc., New York, USA depending on the manufacturer's instructions. The NAc was rapidly taken out of the rat brain for measuring dopamine concentration. Gathered tissues from brain were homogenized in a lysis buffer using a tissue homogenizer and lasted for 5 min on ice. Supernatants were collected from homogenates and stored at −80°C until being used. The dopamine was measured by dopamine ELISA kit (Dopamine Research ELISA, Labor Diagnostika Nord, Germany) depending on the product manual. The optical density was taken at 450 nm using an ELISA reader (MutiRead 400; Authos Co., Vienna, Austria).

### 2.6. [F-18] FDG Micro PET Scan

After test of the behavior, all of the rats fasted for 12–15 h with free access to water in order to increase [F-18] FDG uptake in the rat brain. The animals were warmed with heating pad which was kept at 30°C for 30 min prior to [F-18] FDG injection. Radioactivity in rat brain tissue initially rises quickly following [F-18] FDG injection. When we performed the PET scan, all animals could not move their body, in order to bring the best results. Physiologic monitoring involved determinations of temperature, respiration rate, heart rate, and oxygen saturation.

### 2.7. Voxel-Based Statistical Analysis

Image processing and analysis of the FDG-PET data were conducted using SPM analysis as described previously [1]. Concisely, the brain region was extracted using rectangular masking method. A study-specific template was then composed using all the datasets. The PET data were preprocessed with spatial normalization and smoothing using a 3 mm Gaussian kernel. A voxelwise t-test between the group's datasets was fulfilled using the Statistical Parametric Mapping 5 program (P < 0.05, K > 50). Brain regions were attached to suprathreshold cluster coordinates (medial‐lateral, anterior‐posterior, dorsal‐ventral) using the rat brain atlas.

### 2.8. Statistical Analysis

All results were analyzed using IBM SPSS 23.0 statistics and presented as mean ± standard error of means (SEM). Statistical comparisons were done for the behavioral, immunological studies using the one-way ANOVA followed by the Tukey and the LSD test. Differences with a P value of ≤ 0.05 were considered significant.

## 3. Results

### 3.1. Antidepressant-Like Behavioral Effect of CS

**Figure 3:**
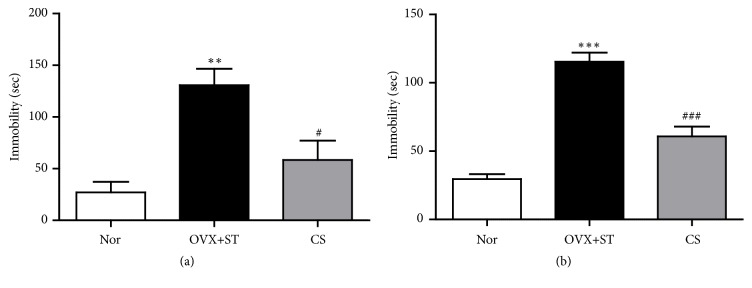
Effects of CS to reduce spent immobility time in the TST and FST. (a) Effect of CS treatment on immobility time in the tail suspension test after restraint stress for 14 consecutive days. (b) Effect of CS treatment on immobility time in the forced swimming test after restraint stress for 14 consecutive days. The data were represented as mean ± standard errors of the mean (SEM). Statistical analysis was conducted using a one-way analysis of variance, followed by post hoc Tukey's test where appropriate, *∗∗∗*, P<0.001; *∗∗*, P<0.01 vs. Nor group, #, P<0.05; ###, P<0.001 vs. OVX+ST group.

The tail suspension test and forced swimming test were used to evaluate behavioral despair. Depressive-like behavior (behavioral despair) was defined as an increase in the time (in seconds) spent immobile. At the beginning of the experiment, the antidepressant-like activity of CS was assessed in ovariectomized rats subjected to IMO by measuring time of immobility during the FST and TST (Figure 3). During the 5 min, the immobility time of the TST was widely different among the groups [F (2,16)=18.826, p<0.05]. Post hoc analysis showed that the immobile duration of the CS group was significantly decreased compared with the OVX+ST group (Figure 3(a)). In the FST, durations of immobility were significantly different among the groups [F (2,16)=41.607, p<0.001]. Increase of immobility time in the TST and FST, reflecting depressive-like behavior, was observed in the OVX-ST group when compared with Nor group. Post hoc analysis exposed that the durations of immobility of the CS group were significantly decreased compared with the OVX+ST group (Figure 3(b)).

### 3.2. Effect of CS on Immobilization-Induced Change of Dopamine Concentrations in the Nucleus Accumbens

**Figure 4:**
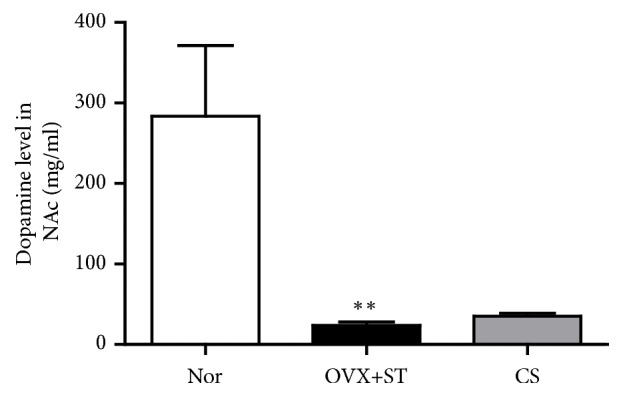
Effects of CS on the alteration of the dopamine level in the NaC. The data were expressed as mean ± standard errors of the mean (SEM). Statistical analysis was conducted using a one-way analysis of variance, followed by post hoc Tukey's test where appropriate, *∗*p<0.01 vs. Nor group.

The result of ELISA suggested that repeated restraint stress for 14 days altered DA concentration in the NAc by 8.12%, compared with rats in the Nor group. In the NAc, the concentration of DA was noticeably reduced in the OVX+ST group, as compared to the Nor (p<0.01; Figure 4). However, CS had no effect on concentration of dopamine in the NAc, as compared to OVX+ST group (p=0.868).

### 3.3. Effect of CS on Immobilization-Induced Change of Estrogen and Oxytocin Concentrations in the Serum

**Figure 5:**
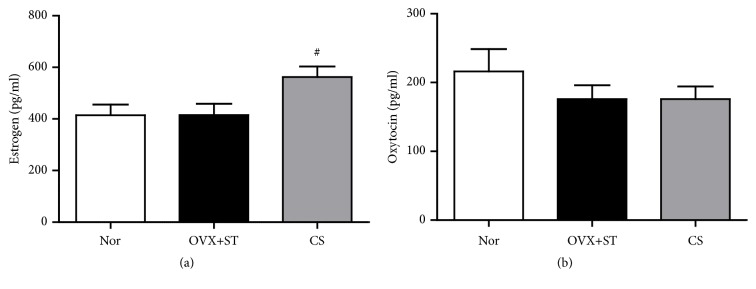
Effect of CS on immobilization-induced change of estrogen and oxytocin concentrations in the serum. The data were expressed as mean ± standard errors of the mean (SEM). Statistical analysis was conducted using a one-way analysis of variance, followed by post hoc Tukey's test where appropriate, #p<0.05 vs. OVX+ST group.

Plasma estrogen level was analyzed in the Nor, OVX+ST, and CS groups. The rats subjected to CS revealed a significantly higher increment of estrogen compared to Nor and OVX+ST (p<0.05; Figure 5(a)). The concentration of oxytocin tended to decrease in the OVX+ST group, as compared to the Nor group. However, CS had no effect on concentration of oxytocin, as compared to OVX+ST group (Figure 5(b)).

### 3.4. Changes in Brain Glucose Metabolism

**Figure 6:**
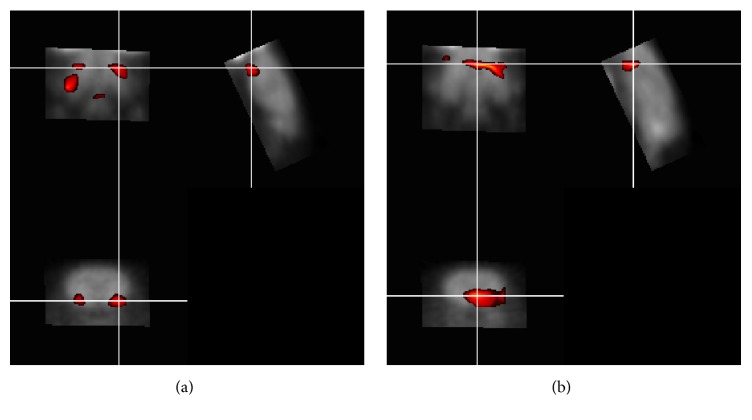
CS changes glucose uptake in the brain. Voxel-wise comparisons between Nor and OVX+ST datasets, (a) OVX+ST and (b) OVX+ST+ CS.

In vivo analysis, the brain glucose metabolism in live rat was conducted using positron emission tomography (Figure 6). We indicated that the effect of CS accords with improving the brain glucose uptake/metabolism through FDG-PET imaging. The study indicated that the uptake of FDG in cortex of the Nor group was markedly increased compared with OVX+ST group (Figure 6(a), P<0.05). The glucose metabolism of the Nor group in the cortex was noticeably increased when compared with OVX+ST+ CS group (Figure 6(b), P<0.05). Glucose metabolism was substantially increased in the cortex of rats from the OVX+ST+ CS compared to the OVX+ST group (Figure 6(b), P<0.05).

## 4. Discussion

Although the pathophysiology of depression remains veiled, there is a tangible proof for the abnormalities of the neurotransmitter systems in depression. The pharmacological antidepressants have been mainly engaged in targeting the monoaminergic system. The antidepressants have been generally connected with targeting the central monoaminergic system. Tricyclic antidepressants (TCAs) have been mainly substituted for clinical use in most parts of the world by effective antidepressant such as selective reversible inhibitors of monoamine oxidase A (RIMAs), selective-norepinephrine reuptake inhibitors (NRIs), selective serotonin reuptake inhibitors (SSRIs), and specific serotonin noradrenaline reuptake inhibitors (SNRIs). Clinically efficient antidepressants contain drugs that have notable structural variety mostly affecting monoamine reuptake or metabolism. However, sequela rise is comparatively common among both the fragmentary responders and the responders without remission, and the drugs unavoidably produce a variety of undesirable side effects [16]. In the present study, we confirmed the hypothesis that CS improved depressive behavior and increased the level of dopamine in the brain. Moreover, we demonstrated that the antidepressants effects of CS were related with the increase of estrogen in the OVX model of depression. Several studies have suggested that estrogen appears to blunt anxiety symptoms and autonomic reactivity to stress. Additionally, recent studies confirm that estrogen alone may have modest effects as a treatment for major depression [17]. However, CS had no effect on oxytocin in the present study.

The FST and TST are used for measuring changes in stress-evoked behavior in rodents and are important to study neurobiological mechanisms included in antidepressants responses [1]. The observed immobility behavior is likely to be a state of helplessness and depression in humans [18]. Attenuation of immobility time demonstrates an antidepressant effect of treated-drugs. These results are compatible with previous studies indicating that depressive behavior from repeated stress and OVX increased immobility during the FST [1]. Also, administration of CS significantly reduced immobility in the FST and TST. Consistently, our results show that CS decreased immobility time on the FST and TST. CS may affect locomotion of animals. In a pilot or our previous study, we did not observe any differences in locomotor activity in ovariectomized stress animal model. However, we did not assess directly locomotor activity test using the extracts in the present study and cannot exclude the possibility of extracts on locomotion, resulting in reducing immobility of TST and FST. In the future we need to test locomotor activity using open-field test.

The present study demonstrated that a functional neuroimaging technique, [F-18] FDG micro PET, can be used to investigate neural correlates of stress response in rat brain. Our study demonstrated that promoted cortex glucose uptake occurs following treatment of CS, suggesting that CS treatment changes neurotransmitter in the brain. One previous study reported that lessened glucose metabolism of prefrontal cortex was reported to be significantly correlated with depression severity in major depressive disorder people [1]. These results suggest that CS may affect glucose metabolism.

In summary, CS has antidepressive effects by reducing immobility time on TST and FST and increasing estrogen levels in the serum. The study suggests evidence that CS may be useful to test potential new antidepressant drugs.

## Figures and Tables

**Table 1:** Composition of catechins and caffeine of high temperature processed-green tea extract.

Component	Green Tea	HTP
	(% w/w)	(% w/w)
Catechin	29.92	10.41
EGC	8.49	1.38
EC	2.25	0.54
EGCG	15.52	4.40
GCG	0.19	2.79
ECG	3.47	1.29
CG	0.03	0.32
Caffeine	8.13	3.70

Total	38.05	14.11

## Data Availability

The data used to support the findings of this study are available from the corresponding author upon request.
